# Dr. Orin C. Shaw

**Published:** 1915-08

**Authors:** 


**Dr. Orin C. Shaw,** Buffalo 1873, died at his home in Casadaga, June 28. He had practiced in Chatauqua Co. for over 40 years, 25 at Kennedy, and 15 at Cassadaga.

